# Impact of a standardized referral to a community pharmacist-led smoking cessation program before elective joint replacement surgery

**DOI:** 10.18332/tid/101600

**Published:** 2019-02-22

**Authors:** Lauren A. Beaupre, Fadi Hammal, Chrisopher DeSutter, Robert E. Stiegelmar, Edward Masson, Barry A. Finegan

**Affiliations:** 1 Collaborative Orthopaedic Research (CORe), Department of Physical Therapy, University of Alberta, Edmonton, Canada; 2 Department of Anesthesiology and Pain Medicine, University of Alberta, Edmonton, Canada; 3 Collaborative Orthopaedic Research (CORe), Department of Surgery, University of Alberta, Edmonton, Canada

**Keywords:** smoking cessation, surgery, community-based resources

## Abstract

**INTRODUCTION:**

Smokers undergoing total joint replacement (TJR) are more likely to develop infections and be re-admitted than non-smokers. The primary purpose of this study was to evaluate the effectiveness of standardized preoperative referral to a community-based pharmacist-led smoking cessation program compared to usual care for patients undergoing TJR. Secondarily, we evaluated the use of the smoking cessation program.

**METHODS:**

A pre-post quasi-experimental study was conducted at a central intake clinic that prepares approximately 3000 TJR patients annually. Participants were recruited at a mean of 13±11.1 weeks preoperatively and provided informed consent. Participants in the ‘pre’ observational phase (OP) received usual care for smoking cessation. For ‘post’ intervention phase (IP) participants, a referral was sent to a community-based pharmacist-led smoking cessation program. Smoking status was validated on study entry using exhaled carbon monoxide. Participants’ smoking status was re-assessed using self-reported point prevalence abstinence at 6 months post-recruitment.

**RESULTS:**

We enrolled 120/150 (80%) potential OP candidates and 104/286 (36%) potential IP candidates. The groups were similar on study entry; overall, the mean age of participants was 58.7±9.1 years and 103 (47%) were male. They reported medium nicotine dependence with 37±11.6 mean years smoked. At 6 months post-recruitment, 8 (7%) OP participants self-reported 30-day point prevalence abstinence compared to 21 (20%) IP participants (p=0.003). Only 58 (56%) IP participants complied with the pharmacist referral, with 19 (33%) of those seeing the pharmacist reporting point prevalence abstinence at 6 months compared to only 2 (4%) of the 45 participants who did not see the pharmacist (p<0.001).

**CONCLUSIONS:**

Referral to a community smoking cessation program as preoperative standard of care is feasible and can enhance long-term quit rates, but voluntary participation led to low recruitment to the program.

## INTRODUCTION

Patients undergoing total joint replacement (TJR) who are current smokers are at increased risk of postoperative complications, including prosthesis failure, infection, and death^1^. A recent propensity score matched analysis using the National Surgical Quality Improvement Program’s database for the years 2011–2012, found that smoking is associated with higher rates of surgical site infection after TJR and that smokers were three times more likely than non-smokers to be readmitted within 30 days of hospital discharge^2^. From these data, it is clear that every effort should be made to assist smokers in quitting or reducing smoking tobacco prior to elective TJR. Despite the recognition of the problem, a recent survey of orthopaedic surgeons found that although 98% reported that they counselled patients on smoking cessation, almost half spent less than five minutes doing so, and 80% either did not delay surgery or delayed surgery for 3 months or less to allow for smoking cessation^3^.

Quitting smoking is difficult and many patients, despite having significant smoking-related disease, remain smokers at the time of surgery^4^. Further, although patients undergoing TJR are generally smokers of long-term duration, evidence suggests that, with support, the likelihood of smoking cessation in ‘hard core’ smokers is similar to other groups of smokers^5^. Thus, every effort should be made to intervene and offer cessation advice^6^.

In Canada, substantial progress has been made in reducing the prevalence of smoking as evidenced by the latest Canadian Tobacco Alcohol and Drugs data from 2015, which gave an overall current smoking prevalence rate of 13%^7^. However, there is a need to develop and test targeted approaches for tobacco cessation in subpopulations such as those undergoing TJR. Incorporation of smoking cessation interventions into standardized preoperative care protocols for elective orthopaedic surgery has been recommended^8,9^, but has not routinely been performed.

A centralized preoperative assessment clinic, where patients’ initial assessment, preoperative surgical optimization and postoperative follow-up occurs in one physical location, is an optimal and appropriate environment for such work^10^. Patients are scheduled for multiple visits to prepare them for surgery with each of these preoperative visits creating an opportunity to provide information on the importance of preoperative smoking cessation. However, current limited research recommends including smoking cessation as another preoperative activity within the clinic environment^11,12^, which may stretch finite clinic resources and time. Instead, we evaluated creating a process in which patients were actively encouraged to access readily available community-based smoking cessation programs within the patient’s own community.

Thus, the primary purpose of this study was to evaluate the effectiveness of preoperative referral to a community-based smoking cessation program offered to smokers as a standardized component of the preoperative pathway compared to usual preoperative care for smokers undergoing TJR. Secondarily, we evaluated the use of the referral to the smoking cessation program.

## METHODS

### Setting

The Edmonton Bone and Joint Clinic is a centralized TJR outpatient clinic where patients undergo initial assessment, preoperative surgical optimization and postoperative follow-up. The clinic serves over 3000 patients undergoing TJR annually with multiple preoperative assessments that occur within one to six months of surgery; this offered us the opportunity to facilitate participation in a community-based smoking cessation program preoperatively. A review of clinic data during the study design phase revealed that 23% of patients that were offered surgery were current smokers (unpublished data).

### Study design and population

A pre-post quasi-experimental design was used in this real-world pragmatic ‘implementation science’ study. The study had 2 phases, a ‘pre’ observational phase (OP) and a ‘post’ interventional phase (IP). All patients scheduled for TJR who were current smokers (any cigarette smoking in the past 30 days), of age 18 years or older and understood English sufficiently to provide written informed consent, and willing to talk with the study nurse, were eligible for participation. Exclusion criteria included: current major psychiatric disorder, previous suicidal behavior, previous psychotic/mood or psychiatric event associated with smoking cessation and/or active substance use disorder.

All study procedures were approved by the human research ethics board at the University of Alberta (Pro00044725). Data were collected between May 2014 and June 2017 using a password-encrypted touch screen computer programmed using Digivey Survey Suite Pro software (Creoso Corporation, Phoenix, Az. USA).

### Procedures

We enrolled participants at the initial consultation with the surgeon. Based on our ethics approval, smokers were identified by the registration clerk who determined if the patient was willing to consider participating in a smoking cessation study and then informed the study coordinator. The coordinator explained the study and willing participants provided signed informed consent. Participants were asked to respond to a brief questionnaire about their smoking history. Smoking status was validated on study entry using exhaled carbon monoxide (CO) monitor (piCO+, Bedfont Scientific Ltd). The CO breath level of 10 ppm was used as a cut-off point to indicate current smoking status^13^. At 6 months post-recruitment, the participants completed the smoking history questionnaire via a telephone interview to report 7- and 30-day point prevalence abstinence.

#### Observational Phase (OP)

In the initial study phase, participants received the clinic’s usual practice for smoking cessation. Clinical staff (both nurses and surgeons) encouraged patients to consider stop smoking preoperatively. This was not a formal counseling session, but rather a short clinical recommendation that typically took less than 5 minutes by each of the health professionals. The nurses offered a referral to a provincial quit smoking hotline if participants were interested.

#### Intervention Phase (IP)

In the IP, participants watched a surgery-specific smoking cessation educational video (https://www.youtube.com/watch?v=Ac84IO4IJkk&feature=you.tube) displayed on a handheld device developed as part of our intervention. The video informed patients of the effects of smoking on wound healing, risk of infection and long-term outcome prior to TJR and detailed the availability of a pharmacist-delivered smoking cessation program in their own community in which the patient could participate. A referral was faxed to a centralized office of a grocery chain that had community pharmacy as part of the services offered within their stores. The preoperative clinic took no further action in promoting smoking cessation other than the referral, and left the smoking cessation intervention at the discretion of the community pharmacy. The program consisted of both behavioral and pharmaceutical interventions of the patient’s preference (i.e. patients did not have to receive pharmaceutical interventions if they preferred only behavioral interventions). The pharmacist and patient determined the number of sessions required on an individual basis. This was an overt decision in the pragmatic research design to evaluate a standardized process within their preoperative clinical pathway with as little interference by the research staff as possible.

The centralized pharmacy office attempted to contact the patient and book a smoking cessation appointment at the pharmacy nearest to his/her home. Three attempts were made to contact the patient before the effort was abandoned. Pharmacists participating in the program underwent smoking cessation training and an additional prescribing course in accordance with standards set by the Alberta Pharmacists’ Association^14^. Following successful completion of the program, the trained pharmacists could independently prescribe all smoking cessation medications, including varenicline^15^. All study participants had universal healthcare coverage with free access to the smoking cessation program provided by the provincial healthcare system.

### Sample size

Sample size calculation was performed for the primary outcome (7-day point prevalence abstinence [PPA]) with 80% power and a two-tailed alpha of 5%. Based on data from the *U.S. Department of Health and Human Services* guidelines published in 2008, a sample size of 90 smokers was needed in each phase to reject the null hypothesis that the abstinence rates for both phases were equal^16^. To account for patient attrition, we aimed to recruit 104 patients in each phase.

### Statistical analysis

Baseline characteristics were compared between the study groups using Student’s t-test and ANOVA for continuous variables, and chi-squared tests for categorical variables. Intention-to-treat analysis was performed in which all subjects were analysed in the group to which they were allocated; all patients who missed follow-up visits were assumed to be still smoking, to provide a conservative estimate of program effect. Comparisons were made between groups for 7- and 30-day point prevalence abstinence at 6 months post-recruitment. Use of the program was also evaluated as was the number of patients who required screening for participation in each phase. Analysis was performed using the Statistical Package for the Social Sciences (SPSS) version 19.0 (IBM SPSS, Armonk, NY).

## RESULTS

**Figure 1:**
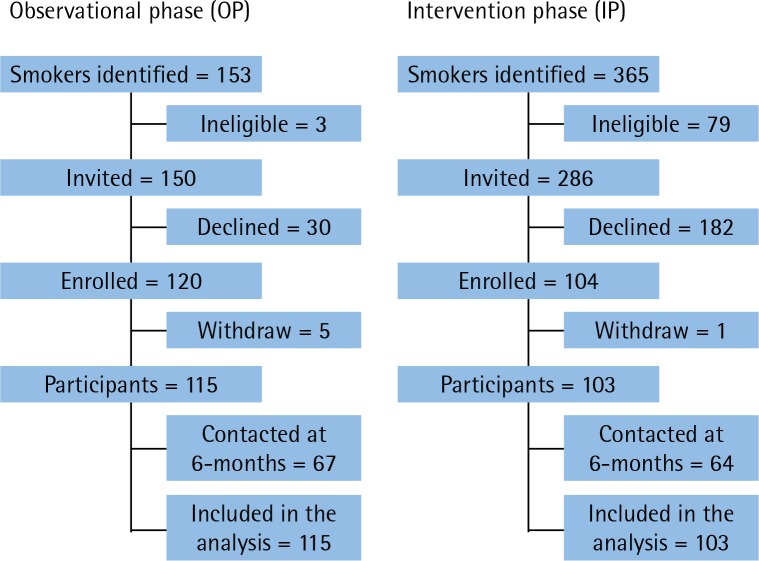
Flowchart of participation by group allocation

We enrolled participants at the initial consultation with the surgeon, which occurred a mean of 13±11.1 weeks preoperatively. The OP started in June 2014 and completed enrollment in Feb 2015; of 150 smokers willing to talk to the study nurse, 120 (80%) agreed to participate; 5 (4%) subsequently withdrew. The IP started in April 2015 and completed enrollment in June 2016; of 286 smokers willing to talk to the study nurse, only 104 (36%) agreed to participate; 1 (1%) subsequently withdrew (Figure 1). At 6 months post-recruitment, 67 (58%) OP and 64 (62%) IP participants completed the phone call.

The mean age for all participants was 58.7± 9.1 years and 103 (47%) were male. More participants in the IP had heart disease (15% vs 5%; p=0.02) and a digestive problem (47% vs 24%; p<0.001) compared to those in the OP, but otherwise the groups were similar on study entry (Table 1).

**Table 1:** Baseline characteristics by study arm

	*OP (N=115 )*	*IP (N=103 )*	*p*
	*n (%)*		
**Demographics**			
Gender (female)	61 (53.0)	54 (52.4)	0.9
Residence (urban)	80 (69.6)	84 (81.6)	0.04
Age, mean±SD	58.7±9.6	58.7±8.4	1.0
Health variables			
Asthma	8 (7.0)	8 (7.8)	1.0
Pulmonary disease	22 (19.1)	31 (30.1)	0.08
Diabetes	19 (16.5)	23 (22.3)	0.3
Heart disease	6 (5.2)	15 (14.6)	0.02
Hypertension	59 (51.3)	50 (48.5)	0.8
Renal disease	2 (1.7)	5 (4.9)	0.3
Stroke	3 (2.6)	4 (3.9)	0.7
Previous operation	104 (90.4)	97 (94.2)	0.3
BMI, mean±SD	31.4±6.7	32.1±6.8	0.4

**Table:** 

	*Mean±SD*		*p*
**Smoking history**			
Fagerström score	4.3±2.2	4.0±2.2	0.4
Years smoked	37.5±13.4	36.9±11.3	0.7
Pack-year	27.7±16.7	27.8±15.1	1.0
CO level at baseline	16.9±7.7	20.7±9.4	0.001
At least 1 previous quit attempt, n (%)	110 (95.6)	99 (96.1)	0.9
Ever used smoking cessation medication, n (%)	79 (68.6)	81 (78.6)	0.1

Participants were also similar in smoking history with both groups reporting medium nicotine dependence (mean Fagerström nicotine dependence score was 4.3±2.2 and 4.0±2.2, for OP and IP, respectively). Overall, the mean years smoked were 37.0±11.6 with 25.8±17.4 pack-years (Table 1). At least one previous quit attempt had been made by 96% of participants.

At 6 months post-recruitment, 8 (7%) participants in the OP self-reported 7-day point prevalence abstinence compared to 21 (20%) in the IP (p=0.003). The 30-day self-reported point prevalence abstinence was 7 (6%) participants in the OP and 20 (19%) in the IP (p=0.002). Only 58 (56%) IP participants complied with the pharmacist referral, with 19 (33%) reporting 7- and 30-day point prevalence abstinence at 6 months. In comparison, of those in IP who did not see the pharmacist, only 2 (4%) reported 7-day point prevalence abstinence with only 1 (2%) reporting 30-day point prevalence abstinence (p<0.001).

## DISCUSSION

In a busy preoperative clinic for TJR patients, a simple referral process to a community-based pharmacist-led smoking cessation program substantially improved smoking cessation at 6 months post-recruitment compared to usual preoperative practice. However, voluntary participation required substantially more screening to enroll willing participants for the active smoking cessation program. While 80% of eligible OP candidates joined the study when offered the ‘passive’ standard of care for smoking cessation, less than 40% of eligible IP candidates agreed to participate when the smoking cessation approach was more structured. Further, of those who agreed to participate in the IP, just over half (56%) of participants followed through despite repeated attempts by the centralized pharmacy office to set up the appointment. Those who saw the pharmacist were significantly more likely to succeed in smoking cessation than those who did not follow through with the referral.

Using pre-admission clinics for preoperative smoking cessation has been evaluated by us^10^ and others^11,12^; all found that active smoking cessation processes could be effectively implemented in these settings. Despite these findings, recent guidelines still recommend that opportunities are needed to improve preoperative smoking cessation^9,17^. Our current study differs from previous evaluations in that it used the pre-admission clinic to identify smokers and facilitate smoking cessation interventions; however clinic staff did not undertake these interventions. Rather, we referred participants to available smoking cessation programs in the community and evaluated if this approach was superior to passive information strategies. This avoided increasing the workload for pre-admission clinic staff.

Our findings align with those of others that passive strategies are ineffective in promoting or prolonging smoking cessation^18^, and support recent reports that community-based pharmacist-led programs are effective^19^. Our province had added benefits; program costs were covered by the provincial healthcare plan and pharmacists were licensed to independently prescribe appropriate pharmaceutical options, including varenicline. This created a simple one-stop option for patients trying to reduce or stop their nicotine intake preoperatively. Based on the different results among participants who used the program and those who did not, the program was highly effective.

Thus, we also identified that mandatory participation might be required for smokers based upon how many participants were screened for participation in the IP and the poor compliance of IP participants in attending the smoking cessation program. As smokers are already stigmatized due to smoking denormalization and successful public health efforts to reduce smoking, mandatory participation could be conceived as coercive^20^. But, there is evidence that smokers still have knowledge gaps about smoking and nicotine addiction^21^. Meeting with trained smoking cessation counselors would appear very helpful to discuss risks associated with surgery and smoking.

An ‘opt-out’ model could be adapted for this process. A recent study used an interactive voice response telephone follow-up system that contacted all smokers post-discharge from hospital unless the patient declined permission while in hospital^22^.

Patients refusing or not-responding to the telephone call furthered the ‘opt-out’ option post-discharge. In the case of TJR, a preoperative visit to the community program could be mandatory, but subsequent visits or receipt of active therapy was left to patient discretion, allowing them to ‘opt-out’. This approach is preferable to denying surgery to patients who smoke, a practice already in place in the UK^23^. Not all patients would quit smoking preoperatively, but exposure to an active smoking cessation program could increase preoperative tobacco reduction or cessation.

Initial mandatory preoperative smoking cessation counseling is analogous to preoperative dental screening to reduce postoperative infection risk, which the BJ clinic currently mandates. The evidence for smoking cessation in reducing postoperative complications is much stronger^1^ than that for routine preoperative dental screening^24,25^, but recent consensus work reported that 80% of orthopaedic surgeons still support dental screening^26^. Thus, our suggested approach of mandatory preoperative smoking cessation counseling with an ‘opt-out’ approach should be acceptable and could substantially increase the likelihood of perioperative tobacco reduction or cessation.

### Strengths and limitations

Our study has some notable strengths; we focused on testing a process that could be embedded within a preoperative clinic rather than developing a new smoking cessation program. Our standardized referral encouraged active smoking cessation therapy preoperatively without increasing clinic burden. We used available community resources where trained health professionals have time to work with individuals to assist them in tobacco reduction and cessation. We also used a quasi-experimental design to compare approaches in ‘real-world’ settings as per our implementation science approach. Although focused on the clinic process, we also evaluated patient use of the referral and the impact of using the program.

Our study has some limitations. Our groups were relatively small and losses to follow-up were higher than expected. The ‘intention-to-treat’ analysis assumed that all participants lost to follow-up continued to smoke, so our results are more likely to under-report versus over-report program impact. We only used self-reported abstinence; however others have shown that self-report can produce results similar to CO monitoring^27,28^. We were unable to re-assess smoking cessation preoperatively because time to surgery was shorter than anticipated, but any tobacco reduction or cessation likely carries health benefits, even when not directly related to reducing postoperative complications^29,30^. Our approach could be enhanced by sending the smoking cessation program referral when booking the appointment with the surgeon to increase preoperative time to stop smoking.

Finally, the quasi-experimental design with the participant as the unit of the analysis (rather than taking a program evaluation approach), required voluntary participation for both the study and the smoking cessation program. Our ethics board required that non-study personnel approach potential participants about study involvement, so only those willing to consider study entry were identified by our study team. Further, in this subset, participation was limited, particularly in OP participants, demonstrating the reluctance of smokers to participate in active smoking cessation programs.

## CONCLUSIONS

Within a busy preoperative clinic, a simple referral process to an active community-based smoking cessation program, embedded within the standardized preoperative clinical pathway, was effective in improving smoking cessation relative to usual care. This process, which uses readily available community-based smoking cessation programs, could be easily implemented in other preoperative clinics without additional clinic resources. Making the referral process mandatory, with an ‘opt-out’ following the initial visit, will ensure that current smokers consult a smoking cessation counsellor preoperatively. This will not ensure that all patients stop smoking preoperatively, but will expose all patients to an active smoking cessation program with appropriate education regarding the risk of smoking and postoperative complications.
